# Effects of Stroboscopic Vision on Depth Jump Motor Control: A Biomechanical Analysis

**DOI:** 10.3390/bioengineering11030290

**Published:** 2024-03-20

**Authors:** Kenneth D. Harrison, Christopher J. Dakin, Anne Z. Beethe, Talin Louder

**Affiliations:** 1 School of Kinesiology, Auburn University, Auburn, AL 36849, USA; kdh0077@auburn.edu; 2 Department of Kinesiology and Health Science, Utah State University, Logan, UT 84322, USA; chris.dakin@usu.edu; 3 PEAK Performance, Colby College Athletics, Waterville, ME 04901, USA; abeethe@colby.edu

**Keywords:** sensory integration, landing, plyometric, GRF, electromyography

## Abstract

Researchers commonly use the ‘free-fall’ paradigm to investigate motor control during landing impacts, particularly in drop landings and depth jumps (DJ). While recent studies have focused on the impact of vision on landing motor control, previous research fully removed continuous visual input, limiting ecological validity. The aim of this investigation was to evaluate the effects of stroboscopic vision on depth jump (DJ) motor control. Ground reaction forces (GRF) and lower-extremity surface electromyography (EMG) were collected for 20 young adults (11 male; 9 female) performing six depth jumps (0.51 m drop height) in each of two visual conditions (full vision vs. 3 Hz stroboscopic vision). Muscle activation magnitude was estimated from EMG signals using root-mean-square amplitudes (RMS) over specific time intervals (150 ms pre-impact; 30–60 ms, 60–85 ms, and 85–120 ms post-impact). The main effects of and interactions between vision and trial number were assessed using two-way within-subjects repeated measures analyses of variance. Peak GRF was 6.4% greater, on average, for DJs performed with stroboscopic vision compared to full vision (*p* = 0.042). Tibialis anterior RMS EMG during the 60–85 ms post-impact time interval was 14.1% lower for DJs performed with stroboscopic vision (*p* = 0.020). Vastus lateralis RMS EMG during the 85–120 ms post-impact time interval was 11.8% lower for DJs performed with stroboscopic vision (*p* = 0.017). Stroboscopic vision altered DJ landing mechanics and lower-extremity muscle activation. The observed increase in peak GRF and reduction in RMS EMG of the tibialis anterior and vastus lateralis post-landing may signify a higher magnitude of lower-extremity musculotendinous stiffness developed pre-landing. The results indicate measurable sensorimotor disruption for DJs performed with stroboscopic vision, warranting further research and supporting the potential use of stroboscopic vision as a sensorimotor training aid in exercise and rehabilitation. Stroboscopic vision could induce beneficial adaptations in multisensory integration, applicable to restoring sensorimotor function after injury and preventing injuries in populations experiencing landing impacts at night (e.g., military personnel).

## 1. Introduction

In a dynamic world, effective motor control relies on integrating sensory information [1], a task carried out by the central nervous system (CNS). The fine-tuning of sensory integration enhances motor performance [2]. Developing a deeper understanding of how the CNS integrates sensory inputs is vital for optimizing exercise training and rehabilitation programs, with applications across a variety of sport and clinical populations. Multisensory integration involves the CNS combining inputs from vestibular, visual, and somatosensory systems to estimate body position and movement relative to the external environment. The CNS adjusts the weight of sensory inputs based on factors like movement complexity, environmental features, and signal reliability [3]. For example, during predictable steady-state movements, the CNS may down-weight the contribution of sensory afferents broadly [4]. Also, in response to disruptions in visual or somatosensory input, the CNS may reweight the contribution of individual sensory inputs [5,6].

Landing from a fall or jump requires the CNS to rely on sensory information for generating skeletal muscle force in anticipation of and reaction to large ground reaction forces (GRFs) [7]. GRFs are applied from the ground to the feet during a landing impact, resulting in a distribution of internal stress through the kinetic chain. The distribution of stress applied to various body structures during landing may depend on the anticipatory activation of agonist and antagonist skeletal muscle arising during the fall [8]. Anticipatory muscle activation leads to an increase in active musculotendinous stiffness prior to landing impact. The muscle–tendon complexes of the lower extremity must achieve an appropriate level of stiffness to dampen the energy of impact and distribute stress amongst joint, ligament, and skeletal structures [7]. Multisensory integration may play a role in fine-tuning stiffness prior to impact, which is relevant to both injury prevention and movement optimization [9]. 

The CNS depends heavily on visual cues when forming an internal model of body position and movement [10]. Accordingly, there is interest in understanding the role of continuous visual input on the control of landing. Researchers frequently employ a ‘free-fall’ paradigm to explore the influence of vision on the control of landing [11]. To carry out the paradigm, participants engage in tasks that simulate functional movements such as descending stairs, landing from a jump, or executing cutting movements in sport. Common movements within the paradigm include drop landings and depth jumps (DJs), where individuals self-initiate a drop from an elevated platform onto a landing surface [11]. Drop landings necessitate the stabilization of the body into a static standing pose post-impact. In contrast, DJs involve a quick absorption of the landing impact that is followed immediately by a maximal vertical jump. As a result, drop landings provide deeper insight into neuromuscular patterns relating to the absorption and stabilization of GRFs post-landing. Alternatively, DJs allow for exploration into the capacity of lower-extremity stretch-shortening cycle (SSC) muscle actions. 

Prior research indicates a role of vision in the motor control of DJs, as evidenced by measurable effects on joint kinematics [12], kinetics [13], and muscle activation patterns during both the drop and landing phases [1]. Commonly, researchers utilize a covering to interrupt continuous visual input, creating binary conditions where the DJ is executed either with or without vision. The ecological validity of this approach is limited when considering that the CNS integration of visual input in real-world settings is a dynamic process contingent upon a multitude of factors [14]. Stroboscopic eyewear is a technological innovation that facilitates controlled visual disruption through the cycling of lens opacity (between opaque and transparent) at a constant rate. The technology provides potential for expanding the scope of investigation into the influence of continuous visual input on motor control during DJs. By reducing the reliability of visual information, stroboscopic vision might induce a compensatory up-regulation of other sensory modalities to support the execution of a desired task [4]. 

The existing literature on stroboscopic vision and DJ motor control is limited. In a recent investigation by Kroll et al. [12], female collegiate volleyball players showed a significant decline in reactive strength index (RSI) scores for DJs performed under stroboscopic conditions compared to with full vision. The authors also noted non-significant increases in peak GRF and rate of GRF development (RFD) for DJs performed under stroboscopic conditions. However, it is important to highlight that they did not normalize GRF measures to account for body weight, potentially impacting the validity and comparability of findings [15]. Additionally, evaluating GRF’s measures in isolation presents limitations in drawing inferences regarding neural aspects of motor control [16]. Within motor control, skeletal muscle acts as the final effector, responsive to both spinal and higher-level CNS control [17]. Theoretically, stroboscopic vision could modify visuomotor integration during the performance of the DJ, potentially leading to changes in CNS motor control strategies that manifest through altered patterns of skeletal muscle activation [18,19]. Surface electromyography (EMG) is frequently utilized by researchers to measure muscle activation through skin conductance, providing an indirect method to investigate motor control globally, across the system hierarchy [20]. Consequently, there is a current need for comprehensive investigation, encompassing EMG and body-weight-normalized GRF measures, to thoroughly understand the effects of stroboscopic vision on DJ motor control.

This cross-sectional investigation aimed to evaluate the influence of stroboscopic vision on the motor control and performance of DJs. We hypothesized that performing DJs under during stroboscopic vision would (1) increase the peak GRF, the rate of force development (RFD), and the peak force reduction (PFR) considering that stroboscopic vision was presumed to affect the optimization of leg stiffness upon landing. We also hypothesized that the uncertainty of landing impact prediction would (2) result in an increase in the pre-landing neural activation of lower limb muscles. Additionally, we anticipated that (3) post-landing muscle activation associated with short- (SLR), medium- (MLR), and long-latency responses (LLR) would be attenuated for lower-extremity extensors when DJs were performed with stroboscopic vision. SLRs are believed to be too quick for volitional intervention since they tend to occur between 30 and 60 ms post-landing. Likewise, SLRs are firmly linked to the musculotendinous stiffness achieved pre-landing. Consequently, (3a) the least discernible effect of stroboscopic vision was expected to align with the LLR (85–120 ms post-landing), while (3b) the most prominent effect was expected for the SLR. The results of this investigation might provide evidence of sensorimotor disruption, indicating the potential utility of stroboscopic vision in exercise and rehabilitation. Stroboscopic vision could be especially relevant for improving multisensory integration, with applications ranging from addressing sensorimotor deficits post-anterior cruciate ligament injury [21] to preventing injuries during nighttime military operations, where visual input is less reliable [22].

## 2. Materials and Methods

### 2.1. Participants

An a priori power analysis (*α* = 0.05, *β* = 0.20, *f* = 0.25) was performed using G*Power (version 3.1). The analysis, considering an estimated correlation of *r* = 0.75 among representative measures based on a prior data set from our lab [12], indicated that a sample of 18 participants would provide adequate statistical power to detect the specified effect size. To account for the possibility of attrition, we recruited twenty male and female young adults to volunteer as participants in this investigation (Table 1). To be eligible, participants (a) had to be between 18 and 35 years of age and (b) self-report as physically active, such that they engaged in a minimum of 90 min per week of moderate- to vigorous-intensity exercise. Participants were required to have incorporated jumping, running, and sprinting movements in their regular physical activity routines. Participants were excluded from the investigation if they (a) reported current physical discomfort or an injury impacting their ability to jump, (b) reported having had surgical intervention on the lower limbs or trunk within the past two years, (c) reported having sustained an ACL or lower-extremity ligament injury in the past, (d) reported having had corrective eye surgery in the past year, (e) reported a visual impairment uncorrectable by refraction (e.g., contacts/glasses), or (f) had a history of concussion or seizure. Prior to enrollment, participants were required to provide written consent via an informed consent document approved by the University Institutional Review Board (Protocol #11961, Approved: 17 August 2021).

### 2.2. Procedures

**Figure 1:**
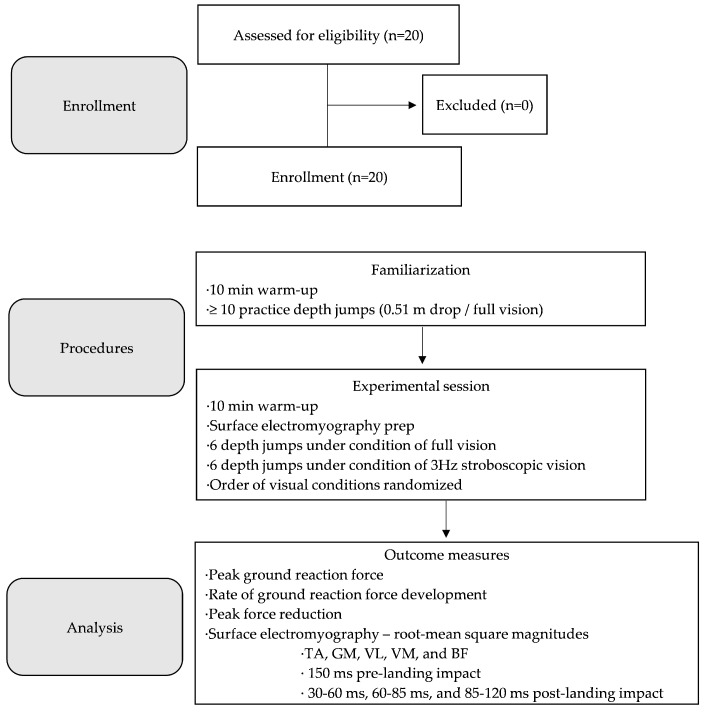
Flow diagram of the investigation protocol. TA = tibialis anterior; GM = medial gastrocnemius; VL = vastus lateralis; VM = vastus medialis; BF = biceps femoris.

The investigation involved a cross-sectional within-subjects design (Figure 1). Participants were invited to a Movement Research Clinic for two testing sessions. Testing sessions were scheduled 48 h apart to ensure adequate recovery time. Participants were asked to refrain from participating in vigorous exercise such as resistance training for 48 h before the first visit and throughout the entire duration of the investigation protocol. Participants were asked to wear athletic attire, specifically gym shorts allowing for exposure of the lower half of the thigh and sport shoes conducive for jumping.

**Figure 2:**
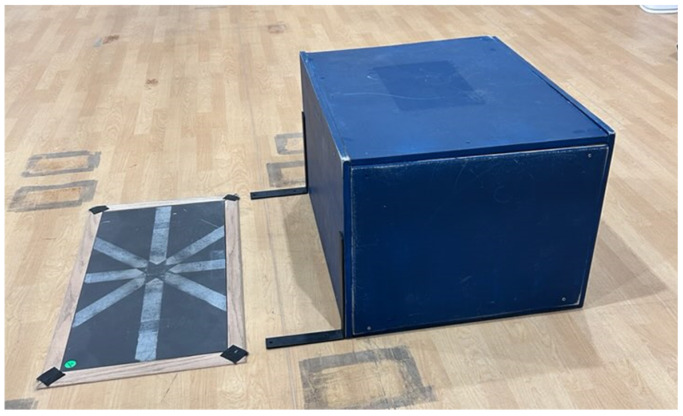
Experimental set-up.

During the first visit, participants first underwent a familiarization of the investigation protocol. Familiarization involved the completion of a 10 min warm-up consisting of stationary cycling for 5 min followed by a standard jumping warm-up as per the National Strength and Conditioning Association (NSCA) [23]. Participants then rested for 3 min while a member of the research team provided a visual demonstration of the DJ technique. Following the demonstration, participants performed a minimum of ten practice DJs with full vision. Practice trials were monitored by a member of the research team, with augmented verbal and visual feedback provided when necessary. The DJ technique involved participants self-initiating a drop from a 0.51 m platform [24,25] to land on a tri-axial force platform (Model FP4080, Bertec Corporation, Columbus, OH, USA) that was recessed to be flush with the laboratory floor and positioned 0.30 m in front of the dropping platform (Figure 2). For each practice trial, participants self-initiated the drop after receiving the following standard verbal cue: “Step forward from the platform with your preferred foot. Land from the drop with both feet impacting the ground simultaneously and perform a maximal jump upwards as quickly and as high as possible” [24,25]. Participants were also instructed to focus their visual gaze on a marker placed 0.30 m in front of the force platform during the drop. Participant arm motion was not restricted, facilitating a jumping technique that more accurately represents the performance of jumping and landing in real-world settings. 

Experimental data were collected during the second visit. Participants first completed the same 10 min warm-up performed during familiarization. During a 10 min rest period, EMG electrodes (Mini Wave Infinity, Cometa, Milan, Italy) were placed over the tibialis anterior (TA), medial gastrocnemius (GM), vastus lateralis (VL), vastus medialis (VM), and biceps femoris (BF) of the leading drop leg. Electrodes were located and attached in accordance with standard recommendations from the SENIAM project (Surface EMG for Non-Invasive Assessment of Muscles; seniam.org). Participants then completed the investigation protocol, consisting of 6 successful DJ trials performed under conditions of full vision (control) and 3 Hz stroboscopic vision (experimental). The order of the conditions was randomized, with all trials performed in each condition before advancing to the second condition. DJ trials were performed using procedures consistent with familiarization. All trials were monitored visually by a member of the research team. Successful trial criteria included landing with both feet simultaneously and performing a maximal jump upwards upon landing with minimal delay (e.g., ground contact time). For each trial, time-series GRF (1000 Hz) and EMG (2000 Hz) signals were time-synched and captured using Vicon Nexus software (Version 2.12, Vicon Motion Systems Ltd., Oxford, UK).

### 2.3. Data Analysis

GRF and EMG signals were exported from Vicon Nexus and processed in MATLAB^®^ (Version R2021a, The Mathworks Inc., Natick, MA, USA). GRF signals were first passed through a low-pass fourth-order recursive Butterworth filter (300 Hz cut-off frequency). GRF signals were then pared to begin at the instant of landing impact using previously described methods [24]. EMG signals were passed through a band-pass fourth-order recursive Butterworth filter (10–500 Hz).

Peak GRF was defined as the maximum value from pared GRF signals. RFD was defined as the ratio of peak GRF to the time interval between DJ landing impact and the expression of peak GRF. PFR was defined as the difference between peak GRF and the first successive local minimum GRF value [25]. Peak GRF, RFD, and PFR were normalized to body weight (BW; [15]).

The pre-activation EMG magnitude for each skeletal muscle was defined as the root mean square (RMS) of EMG data contained within a 150 ms time interval pre-landing impact. The 150 ms pre-landing interval was selected based on the EMG onset latencies (~100 ms) reported previously [11,18,26] and to ensure methodological consistency with recent literature [27,28]. To estimate post-landing EMG magnitude, EMG signals were divided into three time intervals: short-latency response (SLR, 30–60 ms post-landing impact), medium-latency response (MLR, 60–85 ms post-landing impact), and long-latency response (LLR, 85–120 ms post-landing impact). Post-landing EMG magnitude for each skeletal muscle activation during the SLR, MLR, and LLR was defined as the RMS of EMG data within the corresponding time intervals of the SLR, MLR, and LLR. The timing of the SLR, MLR, and LLR used in the present investigation was based on recommendations from prior literature [1,29,30]. While we considered utilizing 30 s windows for the SLR (30–60 ms), MLR (60–90 ms), and LLR (90–120 ms), Taube et al. [29] suggest modifying the MLR to end at 85 ms post-impact to avoid signal contamination from transcortical loops associated with the LLR.

### 2.4. Statistical Analysis

Dependent measures were screened for outliers using a mean ± 3 standard deviation criterion. The screening identified 10 GRF (0.73%) and 90 EMG (0.99%) data points as outliers. Outliers were subsequently removed, and mean imputation was applied to replace the removed data. The normality of dependent measures was confirmed using the Shapiro–Wilk test. The inter-trial reliability of all dependent measures was assessed through intraclass correlation coefficient (ICC) estimates and their corresponding 95% confidence intervals (95% CI). ICC calculations were based on a single measure, absolute agreement, two-way mixed effects model. The interpretation of ICC estimates followed guidelines from Koo and Li [31]. Specifically, values below 0.5 were considered to indicate poor reliability, with values between 0.5 and 0.75 indicating moderate reliability, values between 0.75 and 0.9 indicating good reliability, and values surpassing 0.90 indicating excellent reliability. For all dependent measures, the main effects of and interactions between vision (full vision × stroboscopic vision) and DJ trial number (Trial 1 × Trial 2 × Trial 3 × Trial 4 × Trial 5 × Trial 6) were evaluated from a two-way within-subjects Repeated Measures Analysis of Variance (RMANOVA). Post hoc analysis was conducted upon observation of a significant vision × trial number interaction. Post hoc analysis involved carrying out a one-way RMANOVA, followed by paired *t*-tests. Post hoc analysis conducted upon the observation of a main effect of vision involved follow-up paired *t*-tests. Post hoc analysis conducted upon the observation of a main effect of the trial number involved Bonferroni-adjusted paired *t*-tests. For all dependent measures, Cohen’s *d* effect sizes between stroboscopic and full vision were estimated using mean differences and pooled standard deviations. Stroboscopic and full vision results were collapsed across trial number and correlated using the Pearson *r*. The interpretation of Cohen’s *d* and Pearson *r* values followed established scales from the literature [32,33]. Statistical analyses were performed in RStudio (Version 1.1.456). All hypothesis tests were conducted using an alpha type I error threshold of *p* < 0.05 for determining statistical significance.

## 3. Results 

### 3.1. Inter-Trial Reliability

The inter-trial reliability of GRF and EMG measures is presented in Table 2 and Table 3, respectively.

### 3.2. RMANOVA

#### 3.2.1. Interactions

**Figure 3:**
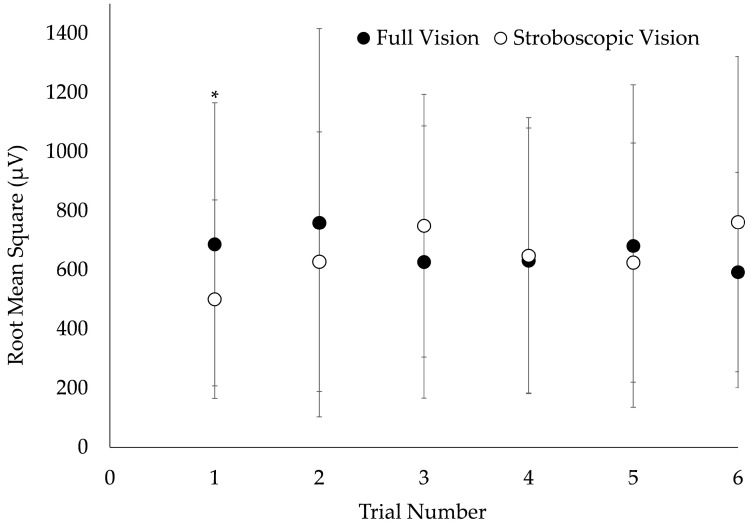
Visual condition × trial number interaction on vastus medialis medium-latency response. Electromyography data were collected from a sample of 20 young adults performing 0.51 m depth jumps under conditions of full and stroboscopic vision. Error bars represent ± 1 standard deviation. * Significantly different from full vision (*p* < 0.05).

A vision × trial number interaction was observed for VM MLR (*F* = 2.4, *p* = 0.037). VM MLR was significantly reduced during stroboscopic vision DJs compared to full vision for trial 1, exclusively (*p* = 0.043; Figure 3). There were no additional vision × trial number interactions on EMG (*F* = 0.2–1.8; *p* = 0.106–0.944) or GRF measures (*F* = 0.4–0.8; *p* = 0.534–0.860).

#### 3.2.2. Main Effects—GRF

A main effect of vision was observed for peak GRF (*F* = 4.5, *p* = 0.035). Post hoc analysis revealed that peak GRF was significantly greater for DJs performed with stroboscopic vision compared to full vision (*p* = 0.042; Table 4). The main effects of vision were not observed for RFD (*F* = 0.6, *p* = 0.439) or PFR (*F* = 1.7, *p* = 0.188). Main effects of the trial number were not observed across all GRF measures (*F* = 0.2–1.6; *p* = 0.152–0.978). 

#### 3.2.3. Main Effects—EMG

Main effects of vision were not observed for pre-activation RMS EMG across all skeletal muscles (*F* = 0.0–3.0, *p* = 0.087–0.902); however, the effect of vision on VL pre-activation RMS EMG approached statistical significance (*F* = 3.0, *p* = 0.087; Table 5). A main effect of trial number was observed for BF pre-activation RMS EMG (*F* = 3.1, *p* = 0.009); however, the post hoc analysis performed using a Bonferroni-adjusted alpha level of 0.003 revealed no significant differences between trials (*p* = 0.010–0.994). The main effects of trial number on pre-activation RMS EMG were not observed for all other skeletal muscles (*F* = 0.5–1.2, *p* = 0.298–0.807).

The main effects of vision (*F* = 0.0–2.5, *p* = 0.117–0.914) and trial number (*F* = 0.6–1.3, *p* = 0.281–0.675) were not observed for SLR EMG across all skeletal muscles. A main effect of vision was observed for TA MLR RMS EMG (*F* = 5.6, *p* = 0.020). Post hoc analysis revealed that TA MLR RMS EMG was lower for DJs performed with stroboscopic vision compared to full vision (*p* = 0.014; Table 5). The main effects of vision were not observed for MLR RMS EMG for all other skeletal muscles (*F* = 0.1–1.0, *p* = 0.319–0.778); however, the effect of vision on BF MLR RMS EMG approached statistical significance (*F* = 3.0, *p* = 0.083; Table 5). The main effects of trial number were not observed for MLR RMS EMG across all skeletal muscles (*F* = 0.7–1.7, *p* = 0.142–0.632). A main effect of vision was observed for VL LLR RMS EMG (*F* = 5.8, *p* = 0.017). Post hoc analysis revealed that VL LLR RMS EMG was lower for DJs performed with stroboscopic vision compared to full vision (*p* = 0.030; Table 5). The main effects of vision were not observed for LLR RMS EMG for all other skeletal muscles (*F* = 0.4–1.6, *p* = 0.206–0.542); however, the effect of vision on VM LLR RMS EMG (*F* = 3.2, *p* = 0.075) and BF LLR RMS EMG (*F* = 3.1, *p* = 0.079) approached statistical significance (Table 5).

## 4. Discussion

This investigation aimed to evaluate how stroboscopic vision affects the motor control of DJs in young adults. Participants performed six trials of the DJ with full vision and six additional trials with 3 Hz stroboscopic vision. The inter-trial reliability of GRF and EMG measures varied from poor to excellent, emphasizing the importance of collecting data across multiple DJ trials. Cohen’s d effect sizes were small for both GRF and EMG measures, likely due to substantial between-subject variability. Despite this, significant differences were observed in peak GRF, TA RMS EMG during the MLR, and VL RMS EMG during the LLR. It is noteworthy that our a priori power analysis was designed to detect significance with a medium Cohen’s d effect size of 0.5. The detection of significance with small effect sizes could be influenced by the moderate to very strong correlations observed between GRF and EMG measures obtained from the stroboscopic and full vision conditions.

Peak GRF averaged 6.4% greater in magnitude, on average, for DJs performed with stroboscopic vision compared to full vision, with a non-significant 3.3% increase in RFD, suggesting a prolonged time to reach peak GRF. A delay in reaching peak GRF might afford more time for post-landing momentum control and the distribution of stress across various musculoskeletal and joint structures. Moreover, the observed difference could imply a mismatch between the stiffness of the limbs achieved pre-landing and the magnitude of landing impact momentum arising from the insufficient visual input available for central processing. Musculotendinous stiffness, crucial for shock absorption, is developed through the dynamic coordination of muscles and joints throughout the kinetic chain [34]. Lower-extremity stiffness is particularly critical during landing impact, directing the application of vertical forces away from vulnerable structures towards skeletal muscles and tendons. While our investigation did not directly estimate lower-extremity stiffness, prior research has linked higher stiffness with increased peak GRF during landing [35]. Although not measured, our investigation suggests a potential stiffness increase pre-landing for DJs performed with stroboscopic vision, as evidenced by greater peak GRFs. Exceeding optimal stiffness upon landing is suggested to elevate the risk of injury to muscles and tendons, while insufficient stiffness may challenge the effective deceleration of joint rotations, heightening the vulnerability of joint structures to injury [7]. It is reasonable to assume that sensorimotor disruption from stroboscopic vision introduces landing impact uncertainty, potentially leading to a preference for loading muscles and tendons with stress through increased stiffness developed at impact, rather than compromising joint control upon landing. 

We hypothesized that observable increases in peak GRF would stem from heightened pre-landing muscle activation. While statistically significant increases in pre-landing RMS EMG were not observed, pre-landing VL and BF RMS EMG increased by an average of 9% and 6%, respectively, for DJs performed with stroboscopic vision, with the increase in pre-landing VL RMS EMG approaching significance (*p* < 0.10). It is important to note that we collected EMG on a subset of superficial skeletal muscles. The complexity of motor control suggests that a broader engagement of muscles may influence the dynamics of lower-extremity stiffness developed in anticipation of landing impact. The present investigation’s design leaves room for uncertainty in interpretation, but it is conceivable that DJs with stroboscopic vision had increased stiffness at landing despite the lack of significant pre-landing RMS EMG observations. Future research could incorporate metrics like vertical or leg stiffness for deeper insights.

Post-landing, there was a significant 14.1% reduction in TA RMS EMG during the MLR for DJs performed with stroboscopic vision. While this could be explained by reciprocal inhibition from the increased potentiation of muscle spindles in ankle plantar flexors, we did not observe a corresponding increase in GM activation. Previous work by Duncan and McDonagh [36] supports the idea that reduced TA activation post-landing may result from muscle spindle potentiation in the plantar flexors. Their examination of post-landing EMG activity in the soleus and GM suggests an increased reliance on involuntary control, with spinal reflex activity associated with early ankle joint rotation during landing [36]. Interestingly, these patterns did not extend to more proximal hip and knee joints, which seemed to be more influenced by pre-programmed responses [36]. The soleus, with a higher density of intrafusal muscle spindle fibers [37], could have initiated a synchronous volley from muscle spindle afferents, contributing to TA inhibition during the MLR, even without observable changes in GM activation. The modulation of the soleus stretch reflex is complex, affected by various factors including α-motor neuron excitability, the γ-motor neuron regulation of intrafusal fiber tension, and pre-synaptic modulation [38]. It is conceivable that the soleus reflex gain increased pre-landing lead to the potentiation of the soleus stretch reflex and the corresponding decrease in TA RMS EMG. However, this speculation necessitates further exploration in future investigations.

Post-landing, there was a significant 11.8% reduction in VL RMS EMG during the LLR for DJs performed with stroboscopic vision. Concurrently, there was a non-significant 9.3% reduction in VM RMS EMG and a non-significant 18.9% increase in BF RMS EMG. These observations suggest a potential stress relaxation response, possibly induced by increased peak GRFs. This response appeared to specifically target the knee extensors through the Ib Golgi tendon spinal reflex arc [39], promoting a greater hamstring-to-quadricep co-activation ratio. The LLR is typically associated with descending voluntary control from the CNS due to the time available for sensory data processing [40]. However, it is important to note that the post-landing time intervals in this investigation were timed to the moment of landing impact. The Ib Golgi tendon spinal reflex arc is considered to be an SLR that is responsive to disturbances in tendon stress. With peak GRF tending to occur about 50 ms post-landing, on average, and the Ib reflex arc having a latency between 20 ms and 50 ms [41], stress relaxation through the Ib reflex arc during the LLR time interval specified in this investigation is a reasonable expectation. 

While this investigation offers valuable insights into the effects of stroboscopic vision on DJ motor control and performance, it is important to acknowledge several limitations. First, we elected to use 3 Hz stroboscopic vision, which, compared to lower frequency settings, is considered less intense. Selecting a higher intensity might have revealed more pronounced effects on GRF and EMG measures. For instance, Kroll et al. [12] found substantially larger effect sizes in various metrics, including RSI scores, jump height, ground contact time, peak GRF, and RFD when utilizing a 1.75 Hz strobe intensity compared to 4 Hz. In our investigation, we opted for a 3 Hz strobe intensity, given that the drop height exceeded that of Kroll et al. [12] by more than 30%. Future investigations could explore different stroboscopic intensities to discern whether a threshold of visual occlusion yields more pronounced effects on neuromuscular activation and foot-ground kinetics. A second limitation pertains to the consideration of true drop height as a potential covariate or moderating variable. Participants may have instinctively lowered themselves more before dropping when performing DJs with stroboscopic vision as a protective measure, a phenomenon observed for increasingly higher drop heights in a prior investigation [24]. Therefore, it is recommended that future investigations incorporate innovative single force platform methods [42] to account for this in statistical analysis. Additionally, we estimated EMG RMS from discrete time intervals (e.g., pre, SLR, MLR, LLR), which may have impacted the robustness of statistical findings. Although these intervals are commonly used to organize EMG signals for inference, they are not rigid and were chosen after reviewing the previous literature [11,18,26]. Adjusting time interval margins or employing alternative techniques (e.g., integrated EMG) might yield different statistical outcomes.

While an a priori analysis was conducted, the inclusion of 20 participants may have limited the statistical power to detect stroboscopic vision effects on DJs. Future research could benefit from a larger or more homogenous sample, with stricter controls on physical activity levels or biological sex. For instance, while this investigation included a mixed-sex sample, we recognize established differences in DJ performance across sexes [43]. Furthermore, future investigations could gain valuable insights by incorporating kinetic and kinematic data at the joint level. The simultaneous analysis of joint-level mechanics in three-dimensional space with EMG data has the potential to enhance the understanding of motor control dynamics, particularly in the context of DJs performed with stroboscopic vision compared to full vision.

## 5. Conclusions

In conclusion, our hypothesis that performing DJs with stroboscopic vision would increase landing impact uncertainty through sensorimotor disruption was supported by several findings. DJs performed in this condition exhibited a 6.4% average increase in peak GRF, indicating a potential development of greater lower-extremity stiffness pre-landing, especially considering the observed trend of increased VL pre-activation. Post-landing, a 14.1% reduction in TA RMS EMG during the MLR suggested reciprocal inhibition from the soleus, despite no significant change in GM RMS EMG. Additionally, an 11.8% reduction in VL RMS EMG during the LLR aligned with trends in decreased VM RMS EMG and increased BF RMS EMG, indicating a heightened stress relaxation response in knee extensors, possibly influenced by the observed peak GRF increase. The observed influence of stroboscopic vision on DJ motor control from the present investigation suggests potential applications, particularly in sensorimotor training and injury prevention. Future research could focus on longitudinal plyometric training with stroboscopic vision, exploring its potential for eliciting sensory re-weighting within the context of multi-sensory integration. In particular, the effectiveness of stroboscopic vision in reducing visual reliance may find applications in restoring sensorimotor function post-anterior cruciate ligament injury and preventing injuries in populations exposed to low light levels, such as military personnel during night-time operations.

## Figures and Tables

**Table 1:** Participant characteristics.

Sex	*n*	Age (Years)
Female	9	21.8 (1.7)
Male	11	22.9 (1.8)

Data are reported as mean (SD).

**Table 2:** Inter-trial reliability of GRF measures.

Measure	Full Vision	Stroboscopic Vision
Peak GRF	0.637 (0.456–0.808)	0.478 (0.286–0.697)
RFD	0.587 (0.401–0.775)	0.643 (0.465–0.812)
PFR	0.619 (0.436–0.797)	0.696 (0.530–0.844)

Ground reaction force (GRF) data were collected from a sample of 20 young adults performing 0.51 m depth jumps under conditions of full and stroboscopic vision. Data are presented as mean ICC estimate (95% confidence interval). GRF = ground reaction force; RFD = rate of force development; PFR = peak force reduction.

**Table 3:** Inter-trial reliability of EMG measures.

Measure	Muscle	Full Vision	Stroboscopic Vision
Pre	VM	0.850 (0.744–0.929)	0.840 (0.728–0.924)
	VL	0.798 (0.667–0.902)	0.942 (0.895–0.974)
	BF	0.611 (0.428–0.791)	0.777 (0.636–0.891)
	TA	0.768 (0.625–0.886)	0.826 (0.708–0.917)
	GM	0.791 (0.656–0.898)	0.836 (0.722–0.922)
SLR	VM	0.826 (0.708–0.917)	0.790 (0.655–0.897)
	VL	0.705 (0.541–0.849)	0.868 (0.772–0.938)
	BF	0.836 (0.723–0.922)	0.692 (0.524–0.842)
	TA	0.388 (0.202–0.624)	0.442 (0.252–0.688)
	GM	0.693 (0.461–0.809)	0.406 (0.220–0.638)
MLR	VM	0.643 (0.465–0.812)	0.666 (0.493–0.826)
	VL	0.784 (0.647–0.894)	0.657 (0.482–0.820)
	BF	0.844 (0.736–0.926)	0.907 (0.835–0.957)
	TA	0.507 (0.318–0.718)	0.505 (0.314–0.718)
	GM	0.666 (0.493–0.826)	0.621 (0.440–0.798)
LLR	VM	0.686 (0.517–0.839)	0.693 (0.527–0.843)
	VL	0.772 (0.630–0.888)	0.736 (0.582–0.867)
	BF	0.882 (0.794–0.945)	0.899 (0.822–0.954)
	TA	0.575 (0.388–0.767)	0.359 (0.177–0.598)
	GM	0.718 (0.559–0.857)	0.608 (0.425–0.789)

Electromyography (EMG) data were collected from a sample of 20 young adults performing 0.51 m depth jumps under conditions of full and stroboscopic vision. Data are presented as mean ICC estimates (95% confidence interval). Pre = pre-activation; SLR = short-latency response; MLR = medium-latency response; LLR = long-latency response; VM = vastus medialis; VL = vastus lateralis; BF = biceps femoris; TA = tibialis anterior; GM = medial gastrocnemius.

**Table 4:** Central tendency and dispersion, and Cohen’s *d* and Pearson *r* results for GRF measures.

Measure	Full Vision	Stroboscopic Vision	Cohen’s *d*	Pearson *r*
Peak GRF (BW)	4.66 (1.26)	4.96 (1.64) *	0.21	0.79
RFD (BW*s-1)	94.27 (49.74)	97.34 (43.72)	0.07	0.86
PFR (BW)	2.28 (1.22)	2.42 (1.44)	0.11	0.87

Ground reaction force (GRF) data were collected from a sample of 20 young adults performing 0.51 m depth jumps under conditions of full and stroboscopic vision. * Significantly different from full vision (*p* < 0.05). RFD = rate of vertical ground reaction force development; PFR = peak force reduction. Data are presented as mean (SD).

**Table 5:** Central tendency and dispersion and Cohen’s *d* and Pearson *r* results for RMS EMG measures.

Measure	Muscle	Full Vision	Stroboscopic Vision	Cohen’s *d*	Pearson *r*
Pre (μV)	VM	376.76 (302.92)	379.30 (340.75)	0.01	0.94
	VL	305.29 (291.25)	334.17 (369.66) ^Ұ^	0.09	0.96
	BF	139.32 (198.07)	147.60 (207.89)	0.04	0.97
	TA	242.79 (123.46)	241.34 (145.00)	−0.01	0.92
	GM	225.11 (114.82)	225.94 (114.77)	0.00	0.98
SLR (μV)	VM	846.16 (630.22)	865.76 (650.52)	0.03	0.95
	VL	613.22 (417.14)	602.75 (485.01)	−0.02	0.94
	BF	336.75 (487.07)	393.72 (533.15)	0.11	0.88
	TA	435.93 (250.43)	438.72 (233.69)	0.01	0.65
	GM	172.12 (153.19)	157.65 (144.89)	−0.10	0.77
MLR (μV)	VM	663.08 (489.32)	654.20 (445.49)	−0.02	0.91
	VL	522.50 (364.44)	553.93 (467.56)	0.08	0.91
	BF	333.83 (604.82)	387.26 (668.96) ^Ұ^	0.08	0.98
	TA	455.44 (306.97)	391.18 (271.92) *	−0.22	0.85
	GM	184.53 (200.94)	179.99 (208.28)	−0.02	0.95
LLR (μV)	VM	673.24 (489.39)	610.51 (430.71) ^Ұ^	−0.14	0.91
	VL	518.23 (402.95)	457.05 (359.28) *	−0.16	0.95
	BF	271.64 (520.39)	323.03 (669.96) ^Ұ^	0.08	0.98
	TA	359.53 (279.72)	341.96 (299.31)	−0.06	0.85
	GM	189.39 (208.99)	212.81 (265.58)	0.10	0.95

Electromyography (EMG) data were collected from a sample of 20 young adults performing 0.51 m depth jumps under conditions of full and stroboscopic vision. * Significantly different from full vision (*p* < 0.05). ^Ұ^ Approached significance (*p* < 0.10). RMS = root mean square; Pre = pre-activation; SLR = short-latency response; MLR = medium-latency response; LLR = long-latency response; VM = vastus medialis; VL = vastus lateralis; BF = biceps femoris; TA = tibialis anterior; GM = medial gastrocnemius. Data are presented as mean (SD).

## Data Availability

The data presented in this study are available on request from the corresponding author. The data are not publicly available due to privacy restrictions.

## References

[1] Helm M., Ritzmann R., Gollhofer A., Freyler K. (2019). Anticipation modulates neuromechanics of drop jumps in known or unknown ground stiffness. PLoS ONE.

[2] Campos J.L., Marusic U., Mahoney J.R. (2022). The intersection of cognitive, motor, and sensory processing in aging: Links to functional outcomes. Front. Aging Neurosci..

[3] Peterka R.J. (2018). Sensory integration for human balance control. Handb. Clin. Neurol..

[4] Kim K.M., Kim J.S., Oh J., Grooms D.R. (2020). Stroboscopic vision as a dynamic sensory reweighting alternative to the sensory organization test. J. Sport Rehabil..

[5] Anson E., Jeka J., Armstrong C.L., Morrow L.A. (2019). Sensory Reweighting: A Rehabilitative Mechanism?. Handbook of Medical Neuropsychology: Applications of Cognitive Neuroscience.

[6] Kim K.M., Kim J.S., Grooms D.R. (2017). Stroboscopic vision to induce sensory reweighting during postural control. J. Sport Rehabil..

[7] Santello M. (2005). Review of motor control mechanisms underlying impact absorption from falls. Gait Posture.

[8] Baudry S., Duchateau J. (2012). Age-related influence of vision and proprioception on Ia presynaptic inhibition in soleus muscle during upright stance. J. Physiol..

[9] Haeufle D.F., Schmortte B., Geyer H., Müller R., Schmitt S. (2018). The benefit of combining neuronal feedback and feed-forward control for robustness in step down perturbations of simulated human walking depends on the muscle function. Front. Comput. Neurosci..

[10] Nashner L., Berthoz A. (1978). Visual contribution to rapid motor responses during postural control. Brain Res..

[11] Liebermann D.G., Goodman D. (2007). Pre-landing muscle timing and post-landing effects of falling with continuous vision and in blindfold conditions. J. Electromyogr. Kinesiol..

[12] Kroll M., Preuss J., Ness B.M., Dolny M., Louder T. (2023). Effect of stroboscopic vision on depth jump performance in female NCAA Division I volleyball athletes. Sports Biomech..

[13] Kamibayashi K., Muro M. (2006). Modulation of pre-programmed muscle activation and stretch reflex to changes of contact surface and visual input during movement to absorb impact. J. Electromyogr. Kinesiol..

[14] Shenton J.T., Schwoebel J., Coslett H.B. (2004). Mental motor imagery and the body schema: Evidence for proprioceptive dominance. Neurosci. Lett..

[15] Mullineaux D.R., Milner C.E., Davis I.S., Hamill J. (2006). Normalization of ground reaction forces. J. Appl. Biomech..

[16] Turns L.J., Neptune R.R., Kautz S.A. (2007). Relationships between muscle activity and anteroposterior ground reaction forces in hemiparetic walking. Arch. Phys. Med. Rehabil..

[17] Enoka R.M. (2008). Neuromechanics of Human Movement.

[18] Santello M., McDonagh M.J., Challis J.H. (2001). Visual and non-visual control of landing movements in humans. J. Physiol..

[19] Shin S.Y., Crapse T.B., Mayo J.P., Sommer M.A., Binder M.D., Hirokawa N., Windhorst U. (2009). Visuomotor integration. Encyclopedia of Neuroscience.

[20] Garcia M.C., Vieira T.M.M. (2011). Surface electromyography: Why, when and how to use it. Rev. Andal. Med. Deporte..

[21] Chaput M., Onate J.A., Simon J.E., Criss C.R., Jamison S., McNally M., Grooms D.R. (2022). Visual cognition associated with knee proprioception, time to stability, and sensory integration neural activity after ACL reconstruction. J. Orthop. Res..

[22] Knapik J.J., Steelman R., Grier T., Graham B., Hoedebecke K., Rankin S., Klug K., Proctor S., Jones B.H. (2011). Military parachuting injuries, associated events, and injury risk factors. Aviat. Space Environ. Med..

[23] Hoffman J.R. (2012). NSCA’s Guide to Program Design.

[24] Louder T., Thompson B.J., Banks N., Bressel E. (2019). A mixed-methods approach to evaluating the internal validity of the reactive strength index. Sports.

[25] Louder T., Thompson B.J., Woster A., Bressel E. (2023). Kinetics of depth jumps performed by female and male national collegiate athletics association basketball athletes and young adults. J. Funct. Morphol. Kinesiol..

[26] Ambegaonkar J.P., Shultz S.J. (2010). Changing filtering parameters affects lower extremity pre-landing muscle activation onset times. Isokinet. Exerc. Sci..

[27] Mrdakovic V., Ilic D.B., Jankovic N., Rajkovic Z., Stefanovic D. (2008). Pre-activity modulation of lower extremity muscles within different types and heights of deep jump. J. Sports Sci. Med..

[28] Han S., Son S.J., Kim H., Lee H., Seeley M., Hopkins T. (2022). Prelanding movement strategies among chronic ankle instability, coper, and control subjects. Sports Biomech..

[29] Taube W., Schubert M., Gruber M., Beck S., Faist M., Gollhofer A. (2006). Direct corticospinal pathways contribute to neuromuscular control of perturbed stance. J. Appl. Physiol..

[30] Waldvogel J., Freyler K., Ritzmann R., Gollhofer A. (2023). Energy transfer in reactive movements as a function of individual stretch load. Front. Physiol..

[31] Koo T.K., Li M.Y. (2016). A guideline of selecting and reporting intraclass correlation coefficients for reliability research. J. Chiropr. Med..

[32] Cohen J. (1988). Statistical Power Analysis for the Behavioral Sciences.

[33] Chan Y.H. (2003). Biostatistics 104: Correlational analysis. Singap. Med. J..

[34] Hughes G., Watkins J. (2008). Lower limb coordination and stiffness during landing from volleyball block jumps. Res. Sports Med..

[35] Shih Y.O., Teng H.L., Powers C. (2019). Lower extremity stiffness predicts ground reaction force loading rate in heel-strike runners. Med. Sci. Sports Exerc..

[36] Duncan A.D., McDonagh M.J. (2000). Stretch reflex distinguished from pre-programmed muscle activations following landing impacts in man. J. Physiol..

[37] Tucker K.J., Türker K.S. (2009). Muscle spindle feedback differs between the soleus and gastrocnemius in humans. Somatosens. Mot. Res..

[38] Ludvig D., Cathers I., Kearney R.E. (2007). Voluntary modulation of human stretch reflexes. Exp. Brain Res..

[39] Lamontagne M., Kennedy M.J. (2013). The biomechanics of vertical hopping: A review. Res. Sports Med..

[40] Cordo P.J. (1990). Kinesthetic control of a multijoint movement sequence. J. Neurophysiol..

[41] Reschechtko S., Pruszynski J.A. (2020). Stretch reflexes. Curr. Biol..

[42] McMahon J.J., Lake J.P., Stratford C., Comfort P. (2021). A proposed method for evaluating drop jump performance with one force platform. Biomechanics.

[43] Polakovičová M., Vavák M., Ollé R., Lehnert M., Sigmund M. (2018). Vertical jump development in elite adolescent volleyball players: Effects of sex and age. Acta Gymnica.

